# Mr. Prior, in Answer to Mr. Chalmers

**Published:** 1807-07

**Authors:** James Prior

**Affiliations:** Assistant Surgeon, Royal Navy. Off Flushing


### *To the Editors of the Medical and Physical Journal.*

Gentlemen,

It is much to be regretted, that the universal Mania of writing should have lately become so general as it is, especially in some members of the medical profession.— When a man, for the puerile vanity of seeing his name in print, makes himself ridiculous by writing *stale remarks on well known subjects*, recommending common applications as new discovered remedies, or attacking the reputation tation of his medical brethren, I cannot but regret their waste of time and paper.

I have lately been perusing the epistle of a Correspondent of yours, a Mr. Chalmers, (whom I have not the pleasure of knowing personally) on pulmonic inflammation; wherein he gravely tells us, that venæsection is absolutely *proper and necessary!* When did Mr. C. make this *important discovery?* Was it in the severe service of a Militia Regiment? Or on the *sanguinary surface of a parade?* Every petty practitioner now aspires to be an author; new hypotheses, new practices, and systems, are daily issuing forth; the press groans under the weight of half-organized medical imaginations, from Theorists without genius, and Authors without common sense. Every writer of a page in a periodical publication has his own peculiar theory and practice; and full of self-importance, issues out his crude, half-formed conceits, as the only true path to proceed in, and then exulting, claims an equality with a Sydenham or a Cullen. I should be extremely sorry to damp that laudable spirit of inquiry, which every professional man ought to possess; my remarks extend only to some petty Letter-mongers, whose genius is confined to a Case, and who must find new origins and terminations, new forms and applications, where experience and reason have distinctly marked the unerring path. The daring flights of Darwin, and the wild imagination of Brown, are in vain attempted to be equalled by the reveries of our new Theory-mongers; though their precepts may not be generally followed, their works will remain lasting testimonials of their genius and abilities.

When Mr. C. penned his production, did he really conceive that the medical world were ignorant of the advantages of venæsection in pulmonic inflammation? or that diluents and acidulated drink were just discovered to be proper? Mr. C. may certainly be only lately enlightened; but I should be extremely sorry indeed, if the youngest tyro in the profession did not know as much on the subject as he has communicated. This gentleman ought to have told us something we did not know. He sets out by saying, "I am nevertheless of opinion, that it is a disease, of which a great proportion of patients die." This I do not believe; for in the majority of cases, where the patient was previously healthy, and perfectly free from all phthisical affection, I have found them terminate successfully by the common means. Again, "This I conceive ceive to be owing, partly to the affection gaining ground before the symptoms sufficiently evince themselves, or, at least, are noticed; and partly to the forbearance and timidity of the practitioner." It is not often the case, I believe, that a pulmonic affection gains any considerable ground before the symptoms evince themselves, for in general they give immediate and distinct warning of what follows; and much seldomer that a medical man, who knows any thing of his profession, would hesitate a moment on the plan he ought to pursue. As to the disease being so often mistaken for typhus, which Mr. C. says is the case, it must be a great oversight of the practitioner; his discrimination must be considerably affected by that mist of prejudice which this gentleman seems so liberally to possess. With respect to the advice on bleeding so profusely, without proper discrimination, it is pernicious; in many cases it may be required, but the patient's constitution, and habit of body, must be attended to, as well as the disease; indiscriminate venæsection has often laid the foundation of a debility from which the patient never recovered. For the health of his Majesty's subjects I hope Mr. C. was not educated in Spain, the doctrines of Sangrado, in that case, might produce some mischief in this country. As to the remainder of this production it requires little comment; it is too common for peculiar remark, and too unimportant to deserve it.

I shall now revert to a subject that has lately attracted my notice, and requires some peculiar and merited animadversions. It is usual for medical men, who may occasionally differ in opinion, to treat their opponents with common decency, if not with civility; how great then was my astonishment and surprise, to see a paper, purporting to be remarks on Mr. Denmark's case of Gastritis, by Mr. Chalmers, the most abusive and unprincipled I ever met with, vilifying not only the professional character of Mr. Denmark, but of the naval medical department in general.

Mr. C. has told them, they do not interest themselves in their duty, and when they do, are incapable of performing it! I was grieved that a man, who from his profession might be supposed to be a gentleman, would so far lay himself open, as to express such an opinion, in such terms. I would ask Mr. Chalmers, whence has this pique (for such it is) against your naval brethren originated? How long has this change of opinion been prevalent in your mind? Was it produced by solitary confinement on the poop poop of a two decker? Or was it in the anguish of resentment, when you were disgracefully turned out of his Majesty's naval service? You, Sir, have been in the medical service of the navy, and you have likewise been turned out of it; a militia regiment has been your refuge, and there concealed, as you think, from the eye of general observation, have dealt out your abusive opinions, and conceited dogmas, with an unsparing hand. These things, Sir, are well known, the friendly shade of obscurity has not been propitious even to such an insignificant person as yourself; it has fallen to me to drag you forth from oblivion, and shew the world why you have such an antipathy against naval surgeons, and naval practice. Contempt and indifference might still have induced me to let you remain unnoticed, but your late attack on Mr. Bellamy, and now on Mr. Denmark, (dictated merely by inveterate malice) require that you should be known; both attacks are equally characteristic of unprincipled meanness and ignorant presumption.

On medical subjects, a difference of opinion should never be productive of abuse or misrepresentation; Mr. Chalmers has been guilty of both, and endeavoured with the greatest diligence to blast the professional character of a gentleman to whom he is unknown, but who has incurred Mr. C's displeasure because he unfortunately belongs to the navy. Mr. C. is the first person who I ever heard declare that the medical department of the navy were incapable of performing their duty. When did Mr. Chalmers ascertain this to be true? What authority, Sir, have you for such an assertion? Do you judge of the navy in general from what you were yourself? I believe this is the case, and in that point of view, Mr. C's self-conviction of incapacity is a liberal acknowledgment.

Medical men who enter into the navy have a great many difficulties, inconveniences, and disagreeables to encounter, which can only be known by those who are in that situation, and which are so connected with the sea service that they cannot be removed; on the other hand, medical men have opportunities and time for study and application, which others ashore do not attend to, who have stronger incentives to attract their passions another way; young men who enter into the army, are in general more induced by the charms of a red coat, than a desire of exercising their profession; the navy has not these incitements, and men of abilities, therefore, will always be numerous in the naval service, notwithstanding the impudent assertion of

Mr.

Mr. C. that they are "incapable of performing their duty." The illiberal and insidious attempt to depreciate their merit, will recoil upon its author; and the public have an opportunity of judging, whether the stings of disappointed ambition have not been more attended to, than the dictates of truth.

With respect to the case of Gastritis itself, I shall leave that principally to Mr. Denmark, allowing myself a few remarks. The great velocity of pulse, while the skin was natural, which Mr. C. seems to doubt, and has almost the assurance to deny, was strictly and literally the case; it was remarked and noted down at the moment. He next complains of the quantity of blood taken not being sufficient, and declaims on "the culpable moderation of the operator." This gentleman should know that it was then, and each time afterwards, taken till syncope was nearly produced.

The exhibition of aromatics, which he seems to speak of as if they were given at first, were not administered before the debility and lowness were extreme, and the patient in a sinking state. Mr. Chalmers, like many others, has his favourite disease, with which every other is mixed and confounded; accordingly, with the greatest consequence and egotism imaginable, he tells us that the disease *was mistaken*; that it *was Pneumonia,* and *not Gastritis!*— This is almost too laughable to require an answer; but if symptoms were not sufficient to inform us, I hope occular demonstration was. On dissection, the lungs were perfectly healthy in every respect, but the stomach and first part of the duodenum were exactly the contrary, having an inflamed and highly erysipelatous appearance, approaching to gangrene. I shall now conclude with an admonition to Mr. Chalmers, to be more cautious and circumspect in his communications for the future, and to refrain from abusing what he cannot equal.

I am, &c.

JAMES PRIOR,

Assistant Surgeon, Royal Navy.

*Off Flushing,*
*March* 25, 1807.

